# Optimal seeding rate enhances seedling quality, mechanical transplanting quality, and yield in hybrid rice

**DOI:** 10.3389/fpls.2024.1427972

**Published:** 2024-06-11

**Authors:** Yuan Feng, Mengzhu Liu, Kunting Wang, Yufei Ling, Qun Hu, Hongcheng Zhang, Haipeng Zhang

**Affiliations:** Jiangsu Key Laboratory of Crop Cultivation and Physiology/Co-Innovation Center for Modern Production Technology of Grain Crops, Research Institute of Rice Industrial Engineering Technology, Yangzhou University, Yangzhou, China

**Keywords:** hybrid rice, blanket seedling cultivation, seeding rate, seedling quality, transplanting quality, grain yield

## Abstract

To determine the appropriate seeding rate for machine-transplanted hybrid rice, field experiments were conducted during 2022–2023 using the hybrid rice variety Huazhe You 210 as the material. Four seeding rate treatments were set up: 40 (T1), 60 (T2), 80 (T3) and 100 g tray^-1^ (T4), to investigate the effects of seeding rate on the seedling quality, transplanting quality, yield formation, and economic benefits of high-quality indica hybrid rice seedlings. The results showed that with increasing seeding rate, the seedling base stem diameter and seedling plumpness of hybrid rice seedlings decreased, but the root entwining force gradually increased, leading to a deterioration in individual seedling quality but an improvement in collective characteristics. As the seeding rate increased, the missing hill rate during mechanical planting of hybrid rice significantly decreased, while the number of seedlings per hill and the damaged seedling rate showed an upward trend. The growth volume of tillers, tillering spikelet rate, and harvest index of hybrid rice in the field showed an overall downward trend with increasing seeding rate, while the accumulation of dry matter initially increased and then decreased. The yield and economic benefits of hybrid rice grains showed an initial increase followed by a decrease with increasing seeding rate, with the highest yield and economic benefits achieved with the T2 treatment. In conclusion, the appropriate seeding rate for machine-transplanted hybrid rice is T2 (60 g tray^-1^), which can maintain good seedling quality and improve transplanting quality, coordinate larger collective growth and appropriate harvest index, contributing to high yield and good economic benefits.

## 1 Introduction

Hybrid rice blanket seedling transplantation is a key technology for promoting the mechanization, simplification, and steady increase in national grain production in China (Zhang and Gong, 2014). This technique typically involves using a high seeding rate combined with a short seedling age method to ensure the formation of a blanket of seedlings while maintaining optimal seedling quality (Cho et al., 2007). It also avoids the reduction in yield caused by seedlings failing to germinate. This technology has become relatively mature in the cultivation of conventional japonica rice. However, when applied to hybrid rice seedling cultivation, challenges arise due to the need for high-density sowing of hybrid rice seeds to form the blanket of seedlings (Huang et al., 2018). Since hybrid rice seeds are costly, this approach can lead to high production costs. Additionally, planting high-density blanket seedlings can result in uneven seedling grabbing and wastage by the transplanting machine, limiting the full potential of hybrid rice’s high-yield advantages. Reducing seed sowing rates prevents the formation of a seedling blanket, significantly impacting the efficiency of subsequent mechanical planting operations (Cheng et al., 2007; Sun, 2013). Developing a method to cultivate a blanket of seedlings has become a technical challenge for hybrid rice blanket seedling transplantation. Current research primarily explores three aspects: variety improvement, controlling seedling age, and adjusting seeding rates. However, due to significant differences in climate resources and planting systems across regions, variety improvement and seedling age control have limitations (Jiang et al., 2016; Chen et al., 2020). Therefore, exploring suitable seeding rates for different varieties and cultivating robust seedlings is the most practical and feasible approach to address this challenge in hybrid rice blanket seedling transplantation.

Appropriate seeding rates for seedling cultivation not only cultivate robust seedlings with increased seedling age elasticity but also reduce the missing hills rate to ensure the quality of mechanical transplantation. Research has shown that lower seeding rates for seedling cultivation optimize individual seedling indicators but deteriorate collective seedling indicators, failing to meet the requirements of mechanical transplantation (Yu et al., 2006). Specifically, there are fewer seedlings per unit area, weak root binding of seedlings, inability to form a seedling blanket, and high rates of missing and leaking hills during machine transplantation. Conversely, higher seeding rates for seedling cultivation result in more seedlings per unit area in the seedling tray, overly dense seedlings, reduced ventilation and light penetration for the seedling group, rapid deterioration of seedling quality, severe planting damage post-transplantation, slow regrowth, and significant constraints on the expression of the variety’s high-yield potential (Sasaki, 2004; Zhang et al., 2018). Thus, determining the appropriate seeding rates for seedling cultivation is crucial for coordinating rice seedling quality and mechanical transplantation quality and is an important prerequisite for achieving high yields in machine-transplanted rice.

Regarding the suitable seeding rates for machine-transplanted rice, conventional japonica rice generally requires a seeding rate of around 120 to 150 g tray^-1^ (Sui et al., 2016; Wang et al., 2016; Huang and Sun, 2017). At this rate, the seeding density is high, the root binding of seedling blocks is good, and the mechanical transplantation quality meets agronomic requirements. However, compared to conventional japonica rice, indica hybrid rice exhibits more significant individual growth advantages (Gong et al., 2014). When the 120 to 150 g tray^-1^ seeding rate for conventional japonica rice is applied to indica hybrid rice, it significantly reduces the quality of indica hybrid rice seedlings (Hu et al., 2017). Therefore, in production, it is often necessary to appropriately reduce the seeding rates for hybrid rice, saving costs while also obtaining better seedling quality. Yao et al. (2009) studied the suitable seeding rates for long-age indica hybrid rice machine transplantation, suggesting that the seeding rate should range from 277.78 to 416.67 g m^-2^ for better coordination of seedling quality and mechanical transplantation quality. Chen et al. (2010) and Jiang et al. (2020) believe that a seeding rate of 60 to 70 g tray^-1^ maintains good seedling quality for machine transplantation, improves mechanical transplantation quality, and coordinates larger group growth and higher harvest indexes, thereby achieving high yields. Li et al. (2014) proposed that the seeding rate for blanket seedling trays of hybrid rice should be between 65 to 80 g tray^-1^. Wang et al. (2019) suggested that the suitable seeding rate for indica hybrid rice should range from 50 to 90 g tray^-1^, at which point seedlings gradually form a blanket.

Although previous studies have extensively researched the suitable seeding rates for machine-transplanted seedlings, the lack of a clear consensus on the ideal seeding rate for hybrid rice is attributed to variations in cultivars, seedling cultivation methods, and regional climatic differences. Additionally, existing research has mainly focused on the relationship between different seeding rates and seedling quality as well as mechanical transplantation quality for various rice varieties. However, there is limited systematic research reporting on the impact of seeding rates on the quality of high-quality indica hybrid rice seedling cultivation, post-transplantation field growth, harvest yields, and the actual economic benefits associated with different seeding rates. Considering this, our study uses the high-quality indica hybrid rice variety Huazheyou 210 as the experimental material. We adopt a 25-day-old seedling age, commonly used in high-yield cultivation practices, to investigate the impact of four different seeding rates on pre-transplantation seedling quality, mechanical transplantation quality, post-transplantation field growth, final yield, and economic benefits. This research aims to provide a theoretical foundation for refining the technical system of high-quality hybrid indica rice seedling cultivation using the blanket seedling machine-transplanting method.

## 2 Materials and methods

### 2.1 Experimental site and materials

The experiment was conducted at the Innovative Experimental Base of Yangzhou University in Shiji Township, Sihong County, Jiangsu Province, China (118°27′E, 33°37′N) during the years 2022–2023. The soil at the site is classified as clay loam, with organic matter content of 27.31 g kg^-1^, total nitrogen content of 1.89 g kg^-1^, available phosphorus content of 32.34 mg kg^-1^, and available potassium content of 85.64 mg kg^-1^ in the 0–20 cm soil layer. The high-quality hybrid rice variety Huazheyou 210 was selected as the experimental material.

### 2.2 Experiment design

To explore the suitable seeding rate for machine-transplanted hybrid rice seedlings, this study included four seeding rates labeled as T1, T2, T3, and T4, corresponding to actual seeding rates of 40 g, 60 g, 80 g, and 100 g, respectively. Hard plastic trays (58 cm × 28 cm × 3 cm) were used for seedling cultivation. The experiments were conducted over two years, with seeding on May 24th and transplanting on June 19th, 25 days after seedling cultivation in the trays. High-speed transplanting machines of the 2ZG-6D (G6) model were used for the transplanting operation, with 2 seedlings per hill, row spacing of 30 cm, and plant spacing of 18 cm. In the field, a slow-release compound fertilizer with resin coating was applied at a rate of 210 kg ha^-1^, with an 80-day release period, containing not less than 15% slow-release nitrogen and a total nutrient content of not less than 51%, in a ratio of N: P_2_O_5_: K_2_O=30: 8: 13, provided by Shandong Maoshi Ecological Fertilizer Co., Ltd. All fertilizers were applied as base fertilization one day before transplanting using mechanical equipment, followed by leveling with a harrow. Subsequent field management during the rice growth stages, including water management and pest control, followed local high-yield cultivation requirements for machine-transplanted rice.

### 2.3 Sampling and measurements

#### 2.3.1 Seedling quality

After 25 days of seedling cultivation, following the measurement method of previous study (Ling et al., 2023), 10 cm × 10 cm seedling blocks were cut from each seeding rate treatment tray. From these blocks, 20 representative seedlings were selected to investigate leaf age, seedling height, seedling base stem diameter, and white root number. After the investigation, all seedlings in the blocks were removed from the seed husks, and the number of seedlings was recorded. The above-ground and below-ground parts of the seedlings were separated, placed in an oven at 105 °C for 30 minutes, dried to a constant weight at 90 °C, and weighed to calculate the dry weight per hundred seedlings for both parts. The seedling plumpness was calculated as the dry weight per hundred seedlings of the above-ground part divided by the seedling height. This measurement was repeated three times.

When measuring the root entwining force of seedlings, one intact seedling block with a 25-day age from each treatment was selected. The moisture content of the seedling block was adjusted to approximately 40%. Both ends of the seedling block were clamped with small wooden boards, and one end was horizontally pulled using an electronic force gauge until the seedling block fractured. The maximum reading of the force gauge at each fracture was recorded as the root entwining force. This measurement was repeated three times for each treatment.

#### 2.3.2 Seedling transplanting quality

Two days after the mechanical transplantation of hybrid rice seedlings using the transplanting machine, a 1.5m long and 1.8m wide area (approximately 50 hills) was selected in each treatment’s field trial area. Following the methodology outlined in previous study (Ling et al., 2021), the number of seedlings planted per hill, the missing hill rate, the damaged seedling rate, and the floating seedling rate were surveyed and recorded. This measurement was repeated three times for each treatment.


(1) DSR(%)=D1/T1×100%

(2) FSR(%)=F1/T1×100%

where, *DSR* is damaged seedling rate (%); *FSR* is floating seedling rate (%); *D_1_* is the number of damaged seedlings; *T_1_* is the total number of seedlings counted; *F_1_* is the number of floating seedlings.

#### 2.3.3 H_2_O_2_ concentration and antioxidant enzyme activity

The H_2_O_2_ concentration and antioxidant enzyme activity were determined by sampling an appropriate amount of fresh samples from different hybrid rice seeding rates treatment, simulating the sampling method during mechanical transplantation. The H_2_O_2_ content was measured using a reagent kit and method provided by Nanjing Jiancheng Bioengineering Institute. The activities of SOD (Superoxide dismutase), CAT (Catalase), APX (Ascorbate peroxidase), and GR (Gluathione reductase) were measured following the methods outlined in previous studies (Giannopolitis and Ries, 1977; Halliwell and Fiyer, 1978; Aebi, 1984).

#### 2.3.4 Tillering dynamics

Starting from 10 days after transplanting, the tiller number of hybrid rice was surveyed. Following the method outlined in previous studies (Tian et al., 2023), three representative observation points were selected for each treatment. At each point, 20 hills were continuously surveyed, totaling 60 hills per treatment. Surveys were conducted every 7 days during the critical growth period of hybrid rice, and the surveys were stopped once the tiller number stabilized until heading stage.

#### 2.3.5 Dry matter accumulation

At the maturity stage, 20 consecutive hills of hybrid rice were randomly surveyed in each plot following the methodology from previous studies (Tian et al., 2023). The average tiller number was calculated based on these surveys. From the average tiller number, three representative rice plants with consistent growth were selected. These selected plant samples were washed, placed in paper bags, and subjected to drying at 105 °C for 30 minutes, followed by drying at 80 °C until a constant weight was achieved. The dry matter mass of the rice plants was then determined at each growth stage.

#### 2.3.6 Grain yield and its components

Before rice harvest, three survey points were selected in each plot to investigate the effective panicle number of rice. Each survey point consisted of 5 rows, with each row containing 20 hills. Based on the calculated average panicle number in each plot, 5 samples were taken (with each hill as one sample) to measure spikelet number per panicle and grain setting rate. After rice harvest, samples of 1000 grains of rice seeds (dry seeds) were weighed three times to calculate the 1000-grain weight. At maturity, each plot harvested an area of 10 m^2^, dried the grains, weighed them, and calculated the actual rice grain yield based on a moisture content of 13.5%.

#### 2.3.7 Economic benefit

During seedling cultivation and transplanting, the number of seedling trays used for each seeding rate treatment was recorded, and the corresponding seedling quantity was calculated. The expenses for cultivating and managing rice per hectare in the field were documented, including costs for agricultural inputs such as pesticides and fertilizers, costs for machine operations during transplanting, labor costs for rice field management, rental fees for field experiments, and expenses for rice harvesting.


(3) $$TP\left(\$\;ha^{-1}\right)=Y\times Ur-\left[(C1\times U1)+(Cs\times Us)+C2+Cl+Co\right]$$

where, *TP* is total profit; *Y* is yield (t ha^-1^); *U_r_* is unit price of rice (the unit price of indica hybrid rice is about 386.42 $ t^-1^); *C_1_* is consumption of seedling trays per hectare; *U_1_* is unit price of seedling cultivation (the unit price of seedling cultivation is 0.4 $ tray^-1^); *C_s_* is consumption of seeds per hectare (kg ha^-1^); *U_s_* is the unit price of Huazheyou 210 (about 13.8 $ kg^-1^); *C_2_* is the cost of seedling transplantation (125 $ ha^-1^); *C_l_* is the labor costs ($ ha^-1^); *C_o_* is the cost of other agricultural inputs including the cost of pesticides and fertilizers, costs for rice field management, rental fees for field experiments, and expenses for rice harvesting, totaling 3126 $ ha^-1^.

### 2.4 Calculation and statistical analyses

Data processing was performed using Excel 2019, while SPSS 26.0 software was used for data analysis. All data were analyzed using a two-way ANOVA including year and treatment. Means were tested by the least significant difference at *p = 0.05* (*LSD0.05*). Origin 9.0 software was used to generate plots.

## 3 Results and analysis

### 3.1 Rice growth period

According to the data in **Table 1**, within the same year of the experiment, there was consistency in the growth period of Huazheyou 210 across different seeding rates. However, some differences were observed between different years during key growth stages. The rice was sown on May 24th in both years, underwent 25 days of seedling cultivation, and was transplanted on June 19th. In 2022, the rice reached the jointing, heading, and maturity stages on July 29th, August 23rd, and October 13th, respectively. In contrast, in 2023, the rice reached the same growth stages on July 27th, August 27th, and October 19th. Comparatively, in 2023, there was a reduction of two days from sowing to jointing, an increase of six days from jointing to heading, and an increase of two days from heading to maturity, resulting in an overall extension of the growth cycle by six days.

**Table 1:** Effects of seeding rate on rice growth period^1)^.

Year	Treatments	ST	TS	JS	HS	MS	ST-JS	JS-HS	HS-MS	WGP
		(m/d)	(m/d)	(m/d)	(m/d)	(m/d)	(d)	(d)	(d)	(d)
2022	T1	5/24	6/19	7/29	8/23	10/13	66	25	52	143
	T2	5/24	6/19	7/29	8/23	10/13	66	25	52	143
	T3	5/24	6/19	7/29	8/23	10/13	66	25	52	143
	T4	5/24	6/19	7/29	8/23	10/13	66	25	52	143
2023	T1	5/24	6/19	7/27	8/27	10/19	64	31	54	149
	T2	5/24	6/19	7/27	8/27	10/19	64	31	54	149
	T3	5/24	6/19	7/27	8/27	10/19	64	31	54	149
	T4	5/24	6/19	7/27	8/27	10/19	64	31	54	149

^1)^ ST, seeding time; TS, transplanting stage; JS, jointing stage; HS, heading stage; MS, maturity stage; WGP, whole growth period (from seeding in the seedbed to harvesting in the paddy field).

### 3.2 Seedling quality

**Figure 1:**
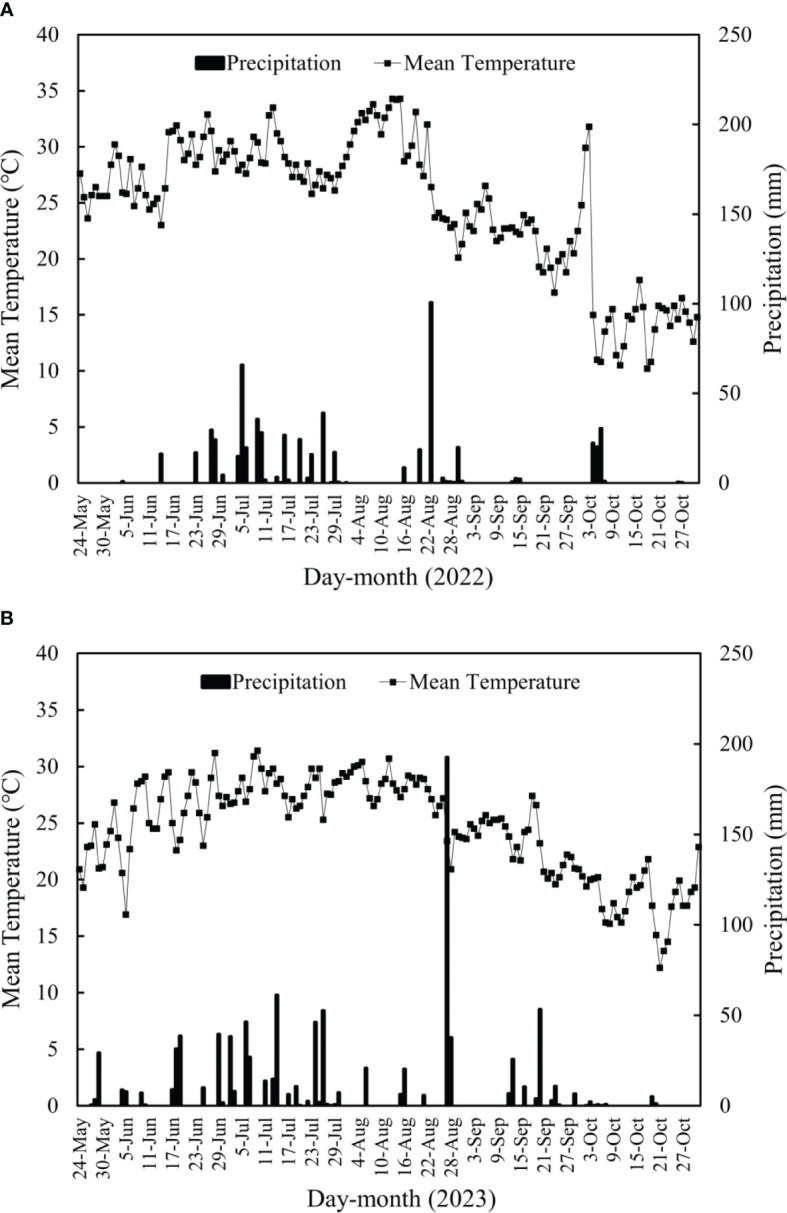
Meteorological data of rice growth season.

The data in **Table 2** indicates a significant impact of different seeding rates on the quality of rice seedlings. Over the course of two years of experimentation, an increase in seeding rate resulted in a decrease in leaf age, seedling base stem diameter, shoot and root dry weight per hundred seedlings, seedling plumpness, and white root numbers, while seedling height and root entwining force showed an increasing trend. Taking the data from 2022 as an example, compared to treatments T2, T3, and T4, treatment T1 saw a reduction in leaf age by 1%, 16.4%, and 26.2%, respectively; seedling base stem diameter decreased by 8%, 18.5%, and 23.5%, respectively; dry weight of shoot for 100 seedlings decreased by 8.1%, 11.8%, and 13.4%, respectively; dry weight of root for 100 seedlings decreased by 20.4%, 29.6%, and 42.8%, respectively; seedling plumpness decreased by 7.6%, 14.5%, and 22.1%, respectively; white root numbers decreased by 5%, 25%, and 25%, respectively; while seedling height increased by 0.2%, 3.1%, and 11.7%, respectively; and root entwining force increased by 44.1%, 75.9%, and 146.5%, respectively. In the 2023 experiment, there were minor differences in some indicators among certain treatments, but the overall trend was similar to that of 2022. Except for seedling base stem diameter, all other indicators showed significant differences between the two years of data, with no significant interaction between year and treatment affecting seedling quality. According to the meteorological data of 2022 and 2023 (**Figure 1**), the average daytime temperature from May 24 to June 19 was 26.97 °C in 2022, significantly surpassing the 2023 average by 11.2%. Higher temperatures correlate positively with faster and more robust rice seedling growth under conditions of uniform artificial irrigation management. Consequently, the quality of rice seedlings in 2022 was conspicuously superior to that observed in 2023. With an increase in seeding rate, there was an improvement in the group indicators of rice seedlings but a decrease in individual indicators.

**Table 2:** Effects of seeding rate on seedling quality^1)^.

Year	Treatments	LA	SH	SD	DWS	DWR	SP	WRN	REF
			(cm)	(mm)	(g)	(g)	(mg cm-^1^)		(kg)
2022	T1	3.06 a	12.82 c	1.62 a	1.86 a	1.52 a	1.45 a	10.0 a	12.25 c
	T2	3.02 a	12.83 c	1.49 a	1.71 b	1.21 b	1.33 b	9.5 ab	17.65 b
	T3	2.55 b	13.21 b	1.32 b	1.64 bc	1.07 bc	1.24 c	7.5 b	21.55 b
	T4	2.25 b	14.32 a	1.24 b	1.61 c	0.87 c	1.13 d	7.5 b	30.20 a
2023	T1	2.61 a	11.54 c	1.56 a	1.77 a	0.97 a	1.54 a	9.5 a	9.10 c
	T2	2.47 ab	11.54 c	1.45 ab	1.63 b	0.86 a	1.41 b	7.0 b	12.30 c
	T3	2.31 bc	12.31 b	1.33 bc	1.60 b	0.80 a	1.30 c	6.0 b	17.35 b
	T4	2.14 c	13.17 a	1.23 c	1.48 c	0.57 b	1.13 d	6.0 b	26.15 a
Year(Y)		**	**	NS	**	**	**	**	**
Treat(T)		**	**	**	**	**	**	**	**
Y×T		NS	NS	NS	NS	NS	NS	NS	NS

^1)^ LA, leaf age; SH, seedling height; SD, seedling base stem diameter; DWS, dry weight of shoot for 100 seedlings; DWR, dry weight of root for 100 seedlings; SP, seedling plumpness; WRN, white root number; REF, root entwining force.

NS, no significance; * and **, significant differences at the 0.05 and 0.01 probability levels, respectively. Values followed by different letters are significantly different at the 0.05 probability level.

### 3.3 Mechanical transplanting quality

**Figure 2:**
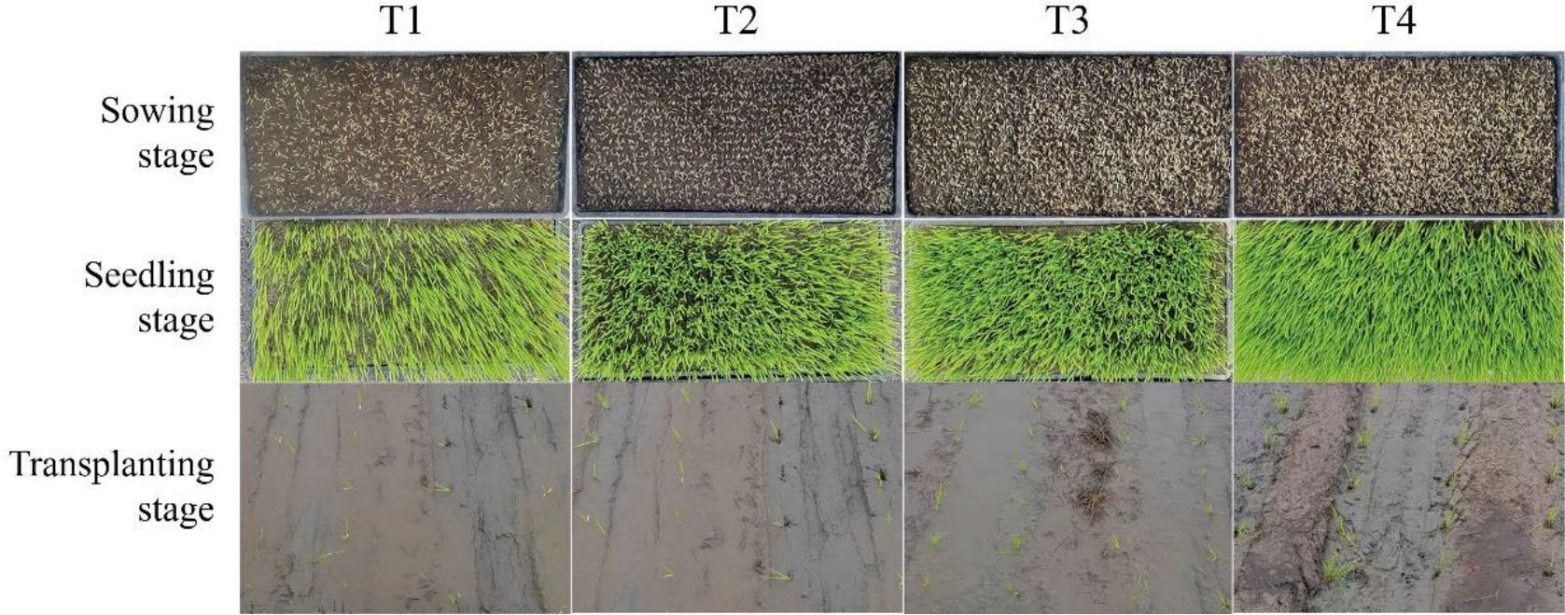
Photographs of different seeding rate at different stage.

From **Table 3**, it is evident that seeding rate has a significant impact on the mechanical transplanting quality of rice. As shown in **Figure 2**, the data from two years of experimentation indicates that as the seeding rate increases, the number of seedlings per hill for mechanical transplanting increases, while the missing hill rate decreases. Treatment T1 shows the lowest uniformity qualification rate, while treatment T4 exhibits the highest damaged seedling rate. However, there is little significant difference in floating seedling rates among the treatments. For example, comparing the data from 2022 with treatment T1, the number of seedlings per hill increased by 38.7%, 59.4%, and 114.2% in the other treatments; meanwhile, the missing hill rates decreased by 5%, 11.1%, and 13.4%, respectively. At this point, the uniformity qualification rate for treatment T1 was 48.3%, significantly lower than the other three seeding rate treatments, while the damaged seedling rate for treatment T4 was 12.8%, significantly higher than the other three seeding rate treatments. However, there was little change in the floating seedling rates. The patterns observed over the two years of data are similar, but the impact of year on indicators such as the number of seedlings per hill, uniformity qualification rate, missing hill rate, damaged seedling rate, and floating seedling rate did not reach a significant level. Different seeding rate treatments had a significant impact on all indicators except for the floating seedling rate, which had no significant impact. The damaged seeding rate was influenced by the interaction between year and treatment, while other indicators were not affected.

**Table 3:** Effects of seeding rate on mechanical transplanting quality^1)^ .

Year	Treatments	NSPH	UQR	MHR	DSR	FSR
			(%)	(%)	(%)	(%)
2022	T1	1.55 d	48.3 b	17.8 a	2.8 c	2.8 a
	T2	2.15 c	83.3 a	12.8 a	3.9 c	3.3 a
	T3	2.47 b	90.0 a	6.7 b	8.9 b	2.2 a
	T4	3.12 a	76.7 a	4.4 b	12.8 a	2.8 a
2023	T1	1.67 c	48.3 b	19.4 a	4.4 b	2.8 a
	T2	2.06 b	75.0 a	13.9 b	5.6 b	3.3 a
	T3	2.34 b	85.0 a	7.2 c	4.4 b	3.3 a
	T4	2.90 a	83.3 a	5.0 c	12.2 a	7.2 a
Year(Y)		NS	NS	NS	NS	NS
Treat(T)		**	**	**	**	NS
Y×T		NS	NS	NS	*	NS

^1)^ NSPH, number of seedlings per hill; UQR, uniformity qualification rate; MHR, missing hill rate; DSR (Equation 1), damaged seedling rate; FSR (Equation 2), floating seedling rate.

NS, no significance; * and **, significant differences at the 0.05 and 0.01 probability levels, respectively. Values followed by different letters are significantly different at the 0.05 probability level.

### 3.4 H_2_O_2_ concentration and antioxidant enzyme activity

The results from **Table 4** indicate that different seeding rates have a significant impact on the H_2_O_2_ concentration and antioxidant enzyme activity of Huazheyou 210 seedlings. The data from two years of experimentation show that, except for APX activity, there were no significant differences between the years in the other indicators. The H_2_O_2_ concentration in the seedlings increases with the increase in seeding rates, and the differences between the treatments reach a highly significant level. Overall, the SOD activity in the seedlings decreases with the increase in seeding rates, with the SOD activity in the T4 treatment significantly lower than the other three seeding rate treatments. The CAT activity in the seedlings also decreases overall with the increase in seeding rates, with small differences between the T1 and T2 treatments, both significantly higher than the T3 and T4 treatments. Similarly, the APX activity in the seedlings decreases overall with the increase in seeding rates. The pattern of GR activity in the seedlings is similar to that of APX activity, decreasing overall with the increase in seeding rates. The increase in seeding rates not only leads to the accumulation of more reactive oxygen species in the seedlings but also reduces the activity of antioxidant enzymes in the seedlings, thereby significantly extending the seedlings’ active regreening period.

**Table 4:** Effects of rice seeding rate on H2O2 concentration and antioxidant enzyme activities of plants^1)^.

Year	Treatments	H_2_O_2_	SOD	CAT	APX	GR
		(mmol·g^-1^)	(U·g^-1^)	(U·g^-1^)	(U·g^-1^)	(U·g^-1^)
2022	T1	4.67 c	33.50 a	412.97 a	2.91 a	150.97 a
	T2	4.75 c	32.37 ab	414.27 a	2.83 b	151.37 a
	T3	4.99 b	32.40 ab	403.20 ab	2.81 b	146.70 b
	T4	5.17 a	31.27 b	397.53 b	2.66 c	141.40 c
2023	T1	4.67 c	33.47 a	418.90 a	2.97 a	157.33 a
	T2	4.80 b	33.60 a	417.03 ab	2.80 b	153.60 b
	T3	4.92 b	33.43 a	404.20 bc	2.74 b	144.40 c
	T4	5.10 a	31.33 b	398.00 c	2.54 c	139.40 d
Year(Y)		NS	NS	NS	*	NS
Treat(T)		**	**	**	**	**
Y×T		NS	NS	NS	**	**

^1)^ SOD, superoxide dismutase; CAT, catalase; APX, ascorbate peroxidase; GR, gluathione reductase. NS, no significance; * and **, significant differences at the 0.05 and 0.01 probability levels, respectively. Values followed by different letters are significantly different at the 0.05 probability level.

### 3.5 Tillering dynamics and tillering spikelet rate

**Figure 3:**
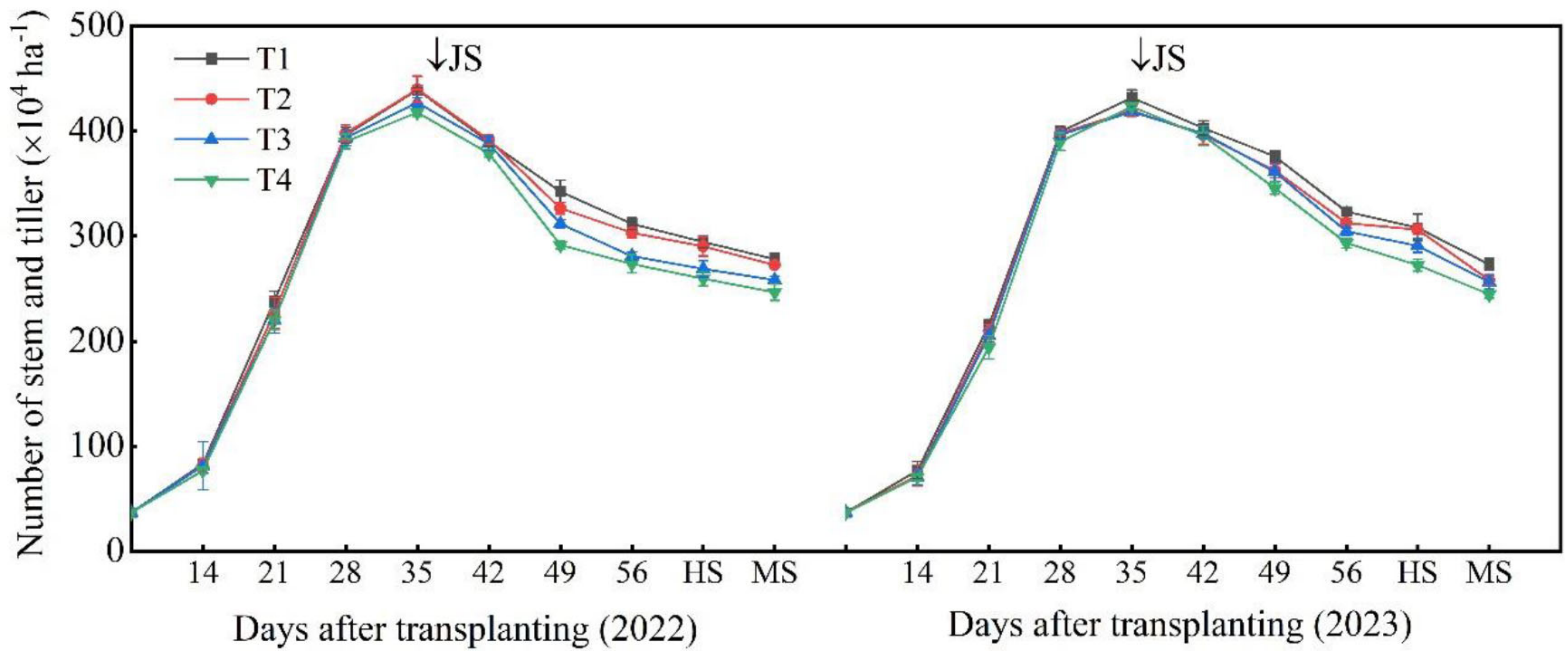
Effect of seeding rate on rice tillering dynamics. JS, jointing stage; HS, heading stage; MS, maturity stage.

From **Figure 3**, it can be observed that different seeding rates do not have a significant impact on the overall growth process of rice, but they do affect the maximum tiller number and effective tiller number of rice. Two years of experimental data show that different seeding rates result in rice starting tillering at approximately 35 days after transplanting, when the total number of tillers in the field reaches its peak. With increasing seeding rates, the total number of tillers in the field decreases. As the growth process progresses, the differences between treatments also gradually diminish until they are nearly negligible. For example, data from 2022 show that there is a small difference in the total number of tillers in the field between T1 and T2 treatments during the heading and maturity stages, and in 2023, there is also a small difference in the total number of tillers in the field between T2 and T3 treatments during the final maturity stage. Despite the diminishing differences in total tiller numbers between treatments, the total number of tillers in the field remains consistently lower for the T4 treatment, while the T1 treatment consistently has the highest total number of tillers in the field at each growth stage.

**Figure 4:**
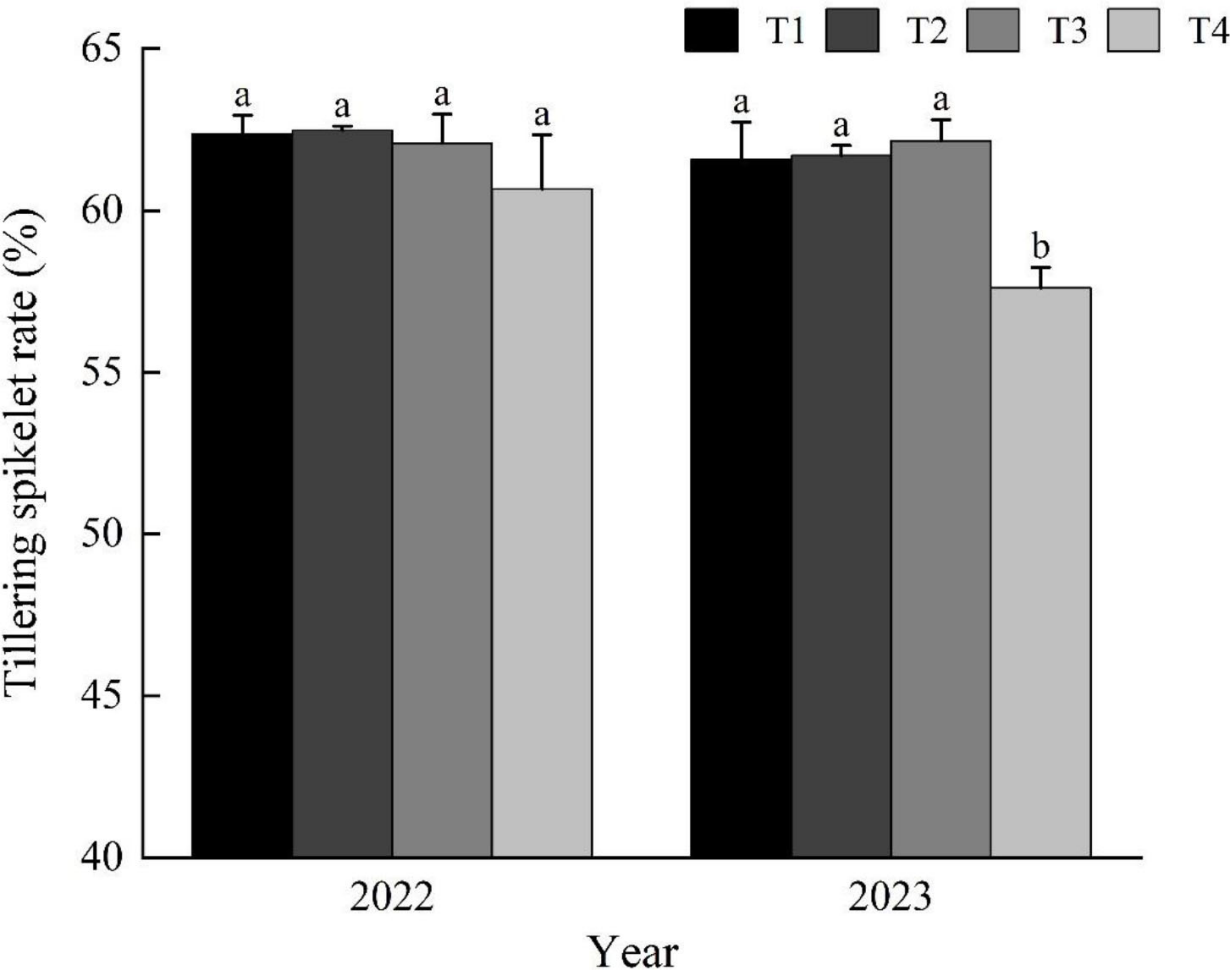
Effect of seeding rate on rice tillering spikelet rate. T1: 40 g tray^-1^; T2: 60 g tray^-1^; T3: 80 g tray^-1^; T4: 100 g tray^-1^.

Furthermore, according to **Figure 4**, seeding rates have a minor but not insignificant impact on the tillering spikelet rate. Data from 2022 show no significant differences in tillering spikelet rates among the four seeding rate treatments, but data from 2023 show that the tillering spikelet rate in the T4 treatment is significantly lower than the other three seeding rate treatments, while there are no significant differences in tillering spikelet rates between the T1, T2, and T3 treatments.

### 3.6 Dry matter accumulation and harvest index

**Figure 5:**
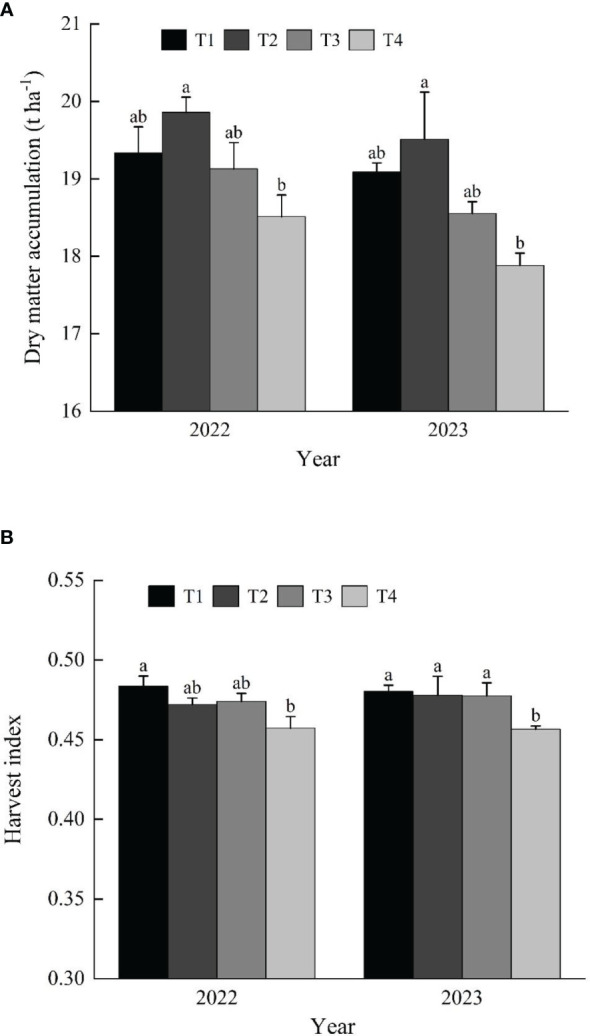
Effects of seeding rate on rice dry matter accumulation **(A)** and harvest index **(B)**. T1: 40 g tray^-1^; T2: 60 g tray^-1^; T3: 80 g tray^-1^; T4: 100 g tray^-1^.

According to the data from **Figure 5A**, there is a trend of increasing then decreasing dry matter accumulation during the maturity stage with increasing seeding rates. Specifically, the T2 treatment exhibits the highest accumulation of dry matter, whereas the T4 treatment demonstrates the lowest. Notably, the dry matter accumulation in the T4 treatment is significantly inferior to that of the T2 treatment, with a notable average reduction of 8.2% over the two years.

As shown in **Figure 5B**, the harvest index shows a decreasing trend with increasing seeding rates. Specifically, there are no significant differences between the T1, T2, and T3 treatments, but the harvest index in the T4 treatment is significantly lower than the other three seeding rate treatments, with reductions of 5.7%, 3.2%, and 3.6%, respectively.

### 3.7 Grain yield and its components

As shown in **Table 5**, seeding rates have a significant impact on the yield of Huazheyou 210. Specifically, over the two-year experiment, the data show a trend of T2 treatment > T1 treatment > T3 treatment > T4 treatment in terms of yield, indicating an initial increase followed by a decrease with increasing seeding rates. The difference between T2 and T1 treatments is minimal, with only a 1% average yield difference over the two years, which is not statistically significant. However, there are significant differences in yield between T2 treatment and T3 treatment as well as T4 treatment, with T2 treatment showing 4.1% and 11% higher yields, respectively.

**Table 5:** Effects of rice seeding rate on grain yield and its components1) .

Year	Treatments	Panicle number	Number of spikelet per panicle	Total number of spikelet	1000-grain weight	Seed-setting rate	Yield
			(×10^4^ ha^-1^)	(×10^4^ ha^-1^)	(g)	(%)	(t ha^-1^)
2022	T1	273.3 a	223.3 ab	61010.9 a	21.4 d	90.4 a	10.81 a
	T2	270.3 ab	226.5 a	61229.4 a	21.5 c	90.7 a	10.84 a
	T3	263.4 b	222.9 ab	58710.2 b	21.7 b	91.4 a	10.48 b
	T4	252.9 c	215.3 b	54452.5 c	22.1 a	91.6 a	9.79 c
2023	T1	268.1 a	216.6 ab	58061.0 a	21.3 c	91.4 a	10.6 a
	T2	260.7 b	223.8 a	58333.6 a	21.3 c	91.3 a	10.78 a
	T3	259.7 b	218.8 ab	56814.9 a	21.5 b	91.9 a	10.24 b
	T4	243.4 c	215.9 b	52528.6 b	22.1 a	91.8 a	9.44 c
Year(Y)		**	NS	**	**	*	**
Treat(T)		**	**	**	**	NS	**
Y×T		NS	NS	NS	NS	NS	NS

^1)^ NS, no significance; * and **, significant differences at the 0.05 and 0.01 probability levels, respectively. Values followed by different letters are significantly different at the 0.05 probability level.

Regarding yield components, seeding rates significantly affect panicle number, number of spikelet per panicle, total number of spikelet, and 1000-grain weight, but not seed setting rate. Although there are small differences in panicle number, number of spikelet per panicle, and total number of spikelet per panicle between certain years and treatments, overall, they decrease with increasing seeding rates. For instance, in 2022, there was a small difference in panicle number between T1 and T2 treatments, but they were significantly higher compared to T3 and T4 treatments by 3.1% to 7%. In terms of number of spikelet per panicle, there was not much difference between T1, T2, and T3 treatments in 2022, but T4 treatment had lower values. However, in 2023, there was no significant difference in number of spikelet per panicle between T1, T3, and T4 treatments, but T2 treatment had significantly higher values. Total number of spikelet showed no significant difference between T1 and T2 treatments in both 2022 and 2023, but they were significantly higher compared to the other two treatments by an average of 4.1% to 11.1%. 1000-grain weight increased with increasing seeding rates. Additionally, there was no significant impact of years on number of spikelet per panicle, and different seeding rate treatments did not significantly affect the seed setting rate. Apart from this, years and different seeding rate treatments had a significant impact on yield and its components, but the interaction between years and treatments did not significantly affect yield and its components.

### 3.8 Correlation analysis

**Figure 6:**
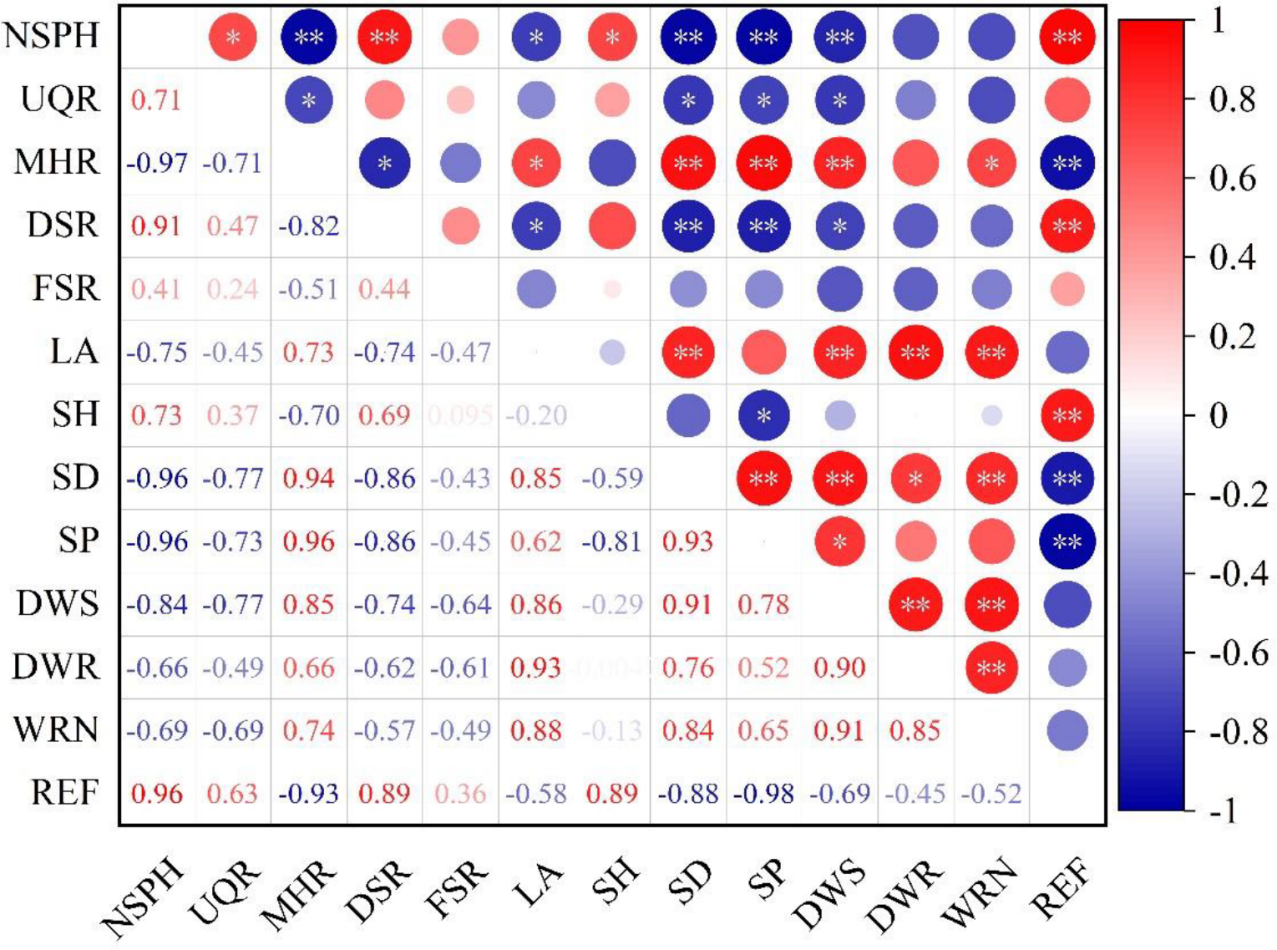
Correlation analysis of seedling quality and mechanical transplanting quality. NSPH, number of seedlings per hill; UQR, uniformity qualification rate; MHR, missing hill rate; DSR, damaged seedling rate; FSR, floating seedling rate; LA, leaf age; SH, seedling height; SD, seedling base stem diameter; SP, seedling plumpness; DWS, dry weight of shoot for 100 seedlings; DWR, dry weight of root for 100 seedlings; WRN, white root number; REF, root entwining force. * and **, significant differences at the 0.05 and 0.01 probability levels, respectively. Values followed by different letters are significantly different at the 0.05 probability level.

**Figure 6** highlights the intricate relationship between the quality of seedlings and the success of mechanical transplanting. Notably, the number of seedlings per hill, the uniformity qualification rate, and the damaged seedling rate exhibit a significant (*P<* 0.05) or highly significant (*P<* 0.01) negative correlation with key metrics such as seedling base stem diameter, seedling plumpness, and the dry weight of the shoot for 100 seedlings. Furthermore, both the number of seedlings per hill and the damaged seedling rate are significantly or highly significantly positively correlated with seedling height and the strength of root entanglement. The missing hill rate shows a significant or highly significant positive correlation with seedling base stem diameter, seedling plumpness, dry weight of the shoot for 100 seedlings, and the number of white roots. Conversely, it exhibits a highly significant negative correlation with root entwining force. Achieving both high-quality seedlings and effective transplanting quality simultaneously is challenging, as robust individual seedling growth often leads to diminished population growth.

**Figure 7:**
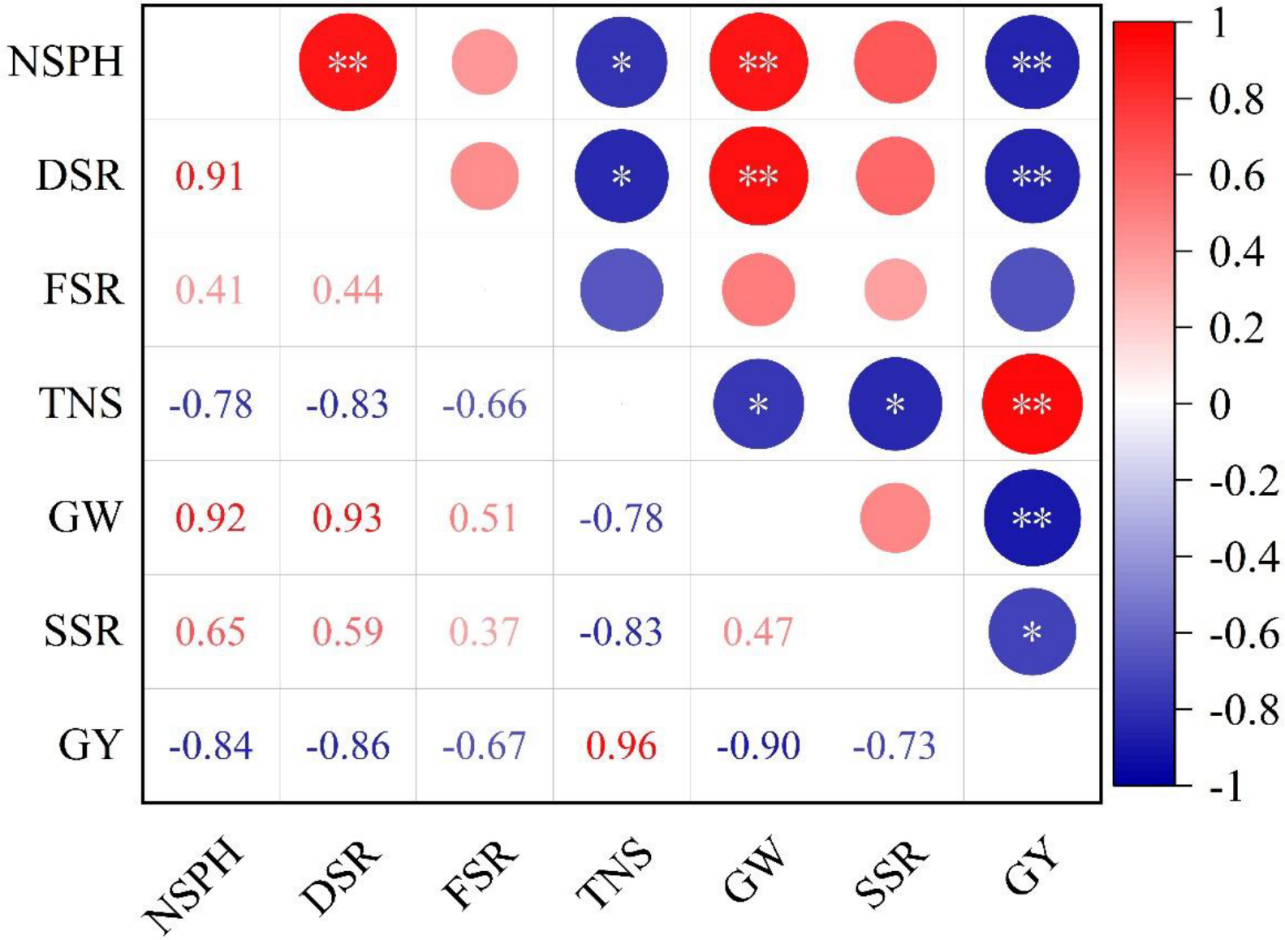
Correlation analysis of mechanical transplanting quality and grain yield and yield components. NSPH, number of seedlings per hill; DSR, damaged seedling rate; FSR, floating seedling rate; TNS, total number of spikelet; GW, 1000-grain weight; SSR, seed-setting rate; GY, grain yield. * and **, significant differences at the 0.05 and 0.01 probability levels, respectively. Values followed by different letters are significantly different at the 0.05 probability level.

**Figure 7** demonstrates the close relationship between yield and its components with mechanical transplanting quality. Specifically, yield is highly significantly negatively correlated with number of seedlings per hill and damaged seedling rate, total number of spikelet is significantly negatively correlated with number of seedlings per hill and damaged seedling rate, while 1000-grain weight is highly significantly positively correlated with number of seedlings per hill. Seed setting rate shows no correlation with any of the indicators.

**Figure 8:**
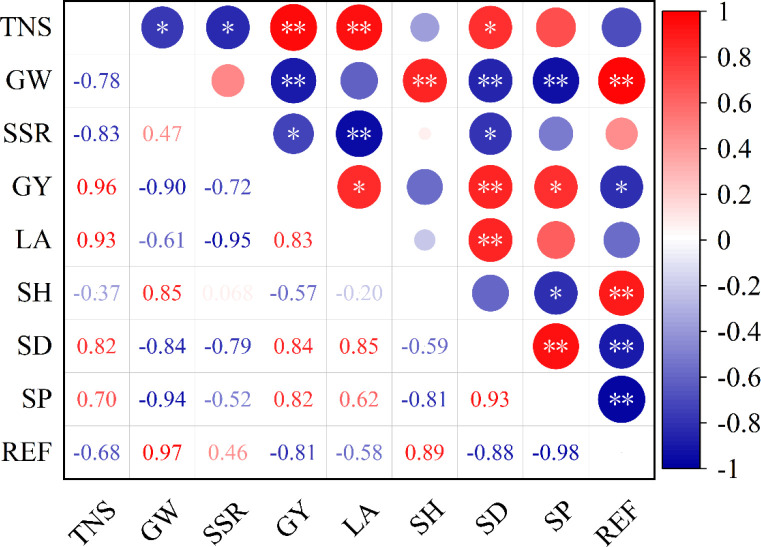
Correlation analysis of seedling quality and grain yield and yield components. TNS, total number of spikelet; GW, 1000-grain weight; SSR, seed setting rate; GY, grain yield; LA, Leaf age; SH, seedling height; SD, seedling base stem diameter; SP, seedling plumpness; REF, root entwining force. * and **, significant differences at the 0.05 and 0.01 probability levels, respectively. Values followed by different letters are significantly different at the 0.05 probability level.

**Figure 8** shows the correlation between seedling quality and yield as well as its components. It shows a significant or highly significant positive correlation between grain yield and the seedling base stem diameter as well as seedling plumpness, indicating that superior seedling quality is conducive to achieving high yields. Besides, Total number of spikelet is significantly positively correlated with seedling base stem diameter. 1000-grain weight is significantly or highly significantly positively correlated with seedling height and root entwining force, while it is significantly or highly significantly negatively correlated with seedling base stem diameter and seedling plumpness.

### 3.9 Economic benefits

The formula for calculating economic benefits has been detailed in the Materials and Methods section, and specific cost calculation data can be found in **Table 6**. Labor costs primarily refer to the supplemental seeding and leveling for missing hills during transplanting. Due to the differences in mechanical transplanting quality among different seeding rates, labor costs are separately compared.

**Table 6:** Effect of rice seeding rate on economic benefits.

Year	Treatments	Consumption of seedling trays	Consumption of seeds	Cost of seedling cultivation and transplantation	Labor costs	Yield benefits	Total profit (Equation 3)
		(tray ha^-1^)	(kg ha^-1^)	($ ha^-1^)	($ ha^-1^)	(t ha^-1^)	($ ha^-1^)
2022	T1	560	22.4	653.6	109.2	10.81	402.8
	T2	390	23.4	551.3	63.8	10.84	517.4
	T3	300	24.0	493.8	35.4	10.48	436.6
	T4	250	25.0	459.7	8.3	9.79	200.3
2023	T1	560	22.4	653.6	109.2	10.6	323.0
	T2	390	23.4	551.3	63.8	10.78	493.5
	T3	300	24.0	493.8	35.4	10.24	343.2
	T4	250	25.0	459.7	8.3	9.44	65.0

**Figure 9:**
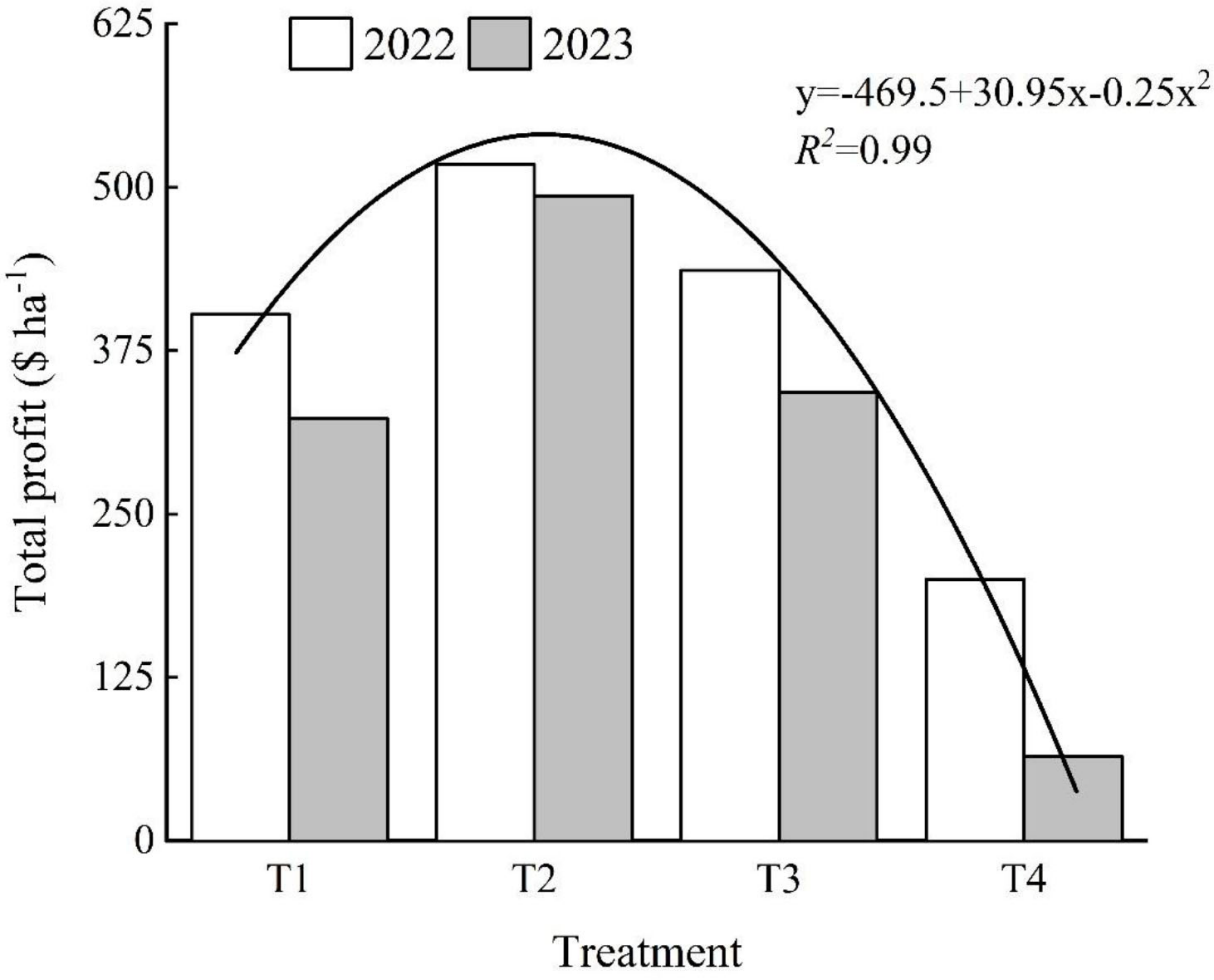
Effect of rice seeding rate on economic benefits.

According to the data in **Figure 9**, economic benefits show a trend of first increasing and then decreasing with increasing seeding rates. Two years of experimental data show that the highest economic benefits are achieved under the T2 treatment, with T1 and T3 treatments yielding similar economic benefits, while the T4 treatment has the lowest economic benefits. Specifically, the T2 treatment achieves good economic benefits by controlling costs while achieving high yields. In contrast, although there is no significant difference in economic benefits between the T1 and T3 treatments, the total cost of seedling cultivation and transplantation is 24.5% higher for T1 than for T3, yet T1 achieves a 3% higher yield, indicating that T1 achieved higher income through higher cost input, while T3 achieved satisfactory income with lower cost input, both yielding considerable profits and good economic benefits. Conversely, despite reducing seedling cultivation and transplanting costs by 42.2%, 20%, and 7.4% compared to the other treatments, T4 performed poorly in terms of yield, resulting in lower profits and poor economic benefits.

Additionally, based on two years of experimental data, a quadratic curve relationship with an *R* ^2^ value of 0.99 was observed between seeding rate (*x*) and economic benefits (*y*) for Huzhou You 210 as follow equation:


$$y=-0.25x^{2}+30.95x-469.5$$

According to this formula, it can be calculated that Huzhou You 210 achieves maximum economic benefits at a seeding rate of approximately 61.9 grams for seedling cultivation.

## 4 Discussion

### 4.1 Effect of seeding rate on seedling quality and mechanical transplanting quality

Seeding rate is a crucial factor influencing the quality of seedlings and mechanical transplanting (Hwang et al., 2021). Previous studies have indicated that with an increase in seeding rate, individual characteristics such as seedling height, root number per seedling, and seedling base stem diameter of hybrid rice decrease, while the number of seedlings per unit area increases, leading to an improvement in population characteristics (Deng et al., 2023). The results of our study confirm this viewpoint, showing that with an increase in seeding rate, traits such as leaf age, seedling base stem diameter, dry weight of shoot and root for 100 seedlings, seedling plumpness, and white root number of Huzhou You 210 exhibit a declining trend, while root entwining force increases, consistent with existing research findings (Yu et al., 2006; Yao et al., 2009; Ou et al., 2019; Wang et al., 2022). However, contrary to previous research results, our study found that seedling height increases with increasing seeding rate. The increase in seeding rate leads to a higher population of seedlings, but the limited growth space within seedling trays intensifies competition among individuals (Zhao et al., 2019), leading to poorer ventilation and light conditions (Zhao et al., 2020). To access better warmth and light resources, individuals exhibit elongation, resulting in increased seedling height but no corresponding increase in aboveground dry weight (González et al., 2003), decreased seedling plumpness, and a slender and fragile morphology. The restricted growth of underground root systems leads to a decrease in white root numbers per seedling, yet the total number of seedlings increases. Under high seeding rates, the overall growth of underground root systems is still greater than under low seeding rates, hence the increase in root entwining force.

Low seeding rates can ensure better individual seedling quality, but in practical production, challenges such as difficulty in forming seedling blankets and high missing hill rates affect mechanical operation quality. Moderately increasing the seeding rate can reduce missing hill rates but is not the sole solution to high missing hill rates. Previous studies have shown that simply increasing seeding rates does not guarantee mechanical transplanting quality (Luo et al., 2009; Shan et al., 2019). The correct approach is to properly allocate seeding rates and field harvesting areas to ensure mechanical transplanting quality. Our study found that with an increase in seeding rate, the number of seedlings per hill increases, missing hill rates decrease, but damaged seedling rates rise, and uniformity qualification rates first rise and then fall, indicating that mechanical transplanting quality does not necessarily improve with increasing seeding rates. Although the number of seedlings per hill increases under high seeding rates, the rise in seedling height leads to increased damaged seedling rates and poorer mechanical transplanting quality. Among all treatments, the T3 treatment exhibited seedling numbers per hill and uniformity qualification rates closest to production needs while controlling damaged seedling rates, making it the best for mechanical transplanting quality.

Cultivating robust seedlings for blanket transplantation requires not only good seedling quality but also ensuring mechanical transplanting quality. Although seedling quality is optimal under T1 seeding rates, mechanical transplanting quality is poor; T4 treatment shows poor seedling quality and unsatisfactory mechanical transplanting quality; whereas T2 and T3 treatments strike a better balance between seedling quality and mechanical transplanting quality.

### 4.2 Effect of seeding rate on grain yield and its components

Seeding rate has a significant impact on the yield components and final yield of rice. Previous studies have suggested that higher seeding rates may decrease the seedling quality, affecting the regrowth vigor of seedlings after transplanting and consequently shortening the vegetative growth period of rice (Hossen et al., 2022; Ling et al., 2023). This, in turn, can impact the panicle formation rate and ultimately lead to reduced yields. The results of this study also support this viewpoint, showing a trend of increasing yields followed by a decrease with increasing seeding rates, with the highest yield observed under the T2 treatment.

Further analysis of yield components revealed that lower seeding rates may lead to an increase in effective panicle number. This could be due to the seeding rate affecting seedling quality, which subsequently influences the regrowth vigor of seedlings post-transplanting. The study also found that after transplanting, the H_2_O_2_ concentration and antioxidant enzyme activity of seedlings under different seeding rates were measured. The results showed an increase in H_2_O_2_ levels with increasing seeding rates, while antioxidant enzyme activity exhibited a decreasing trend. This suggests that under high seeding rates, seedling growth and development may stagnate, while lower levels of antioxidant enzyme activity could prolong the stagnation period of growth and development, thereby affecting seedling growth (Lee et al., 2021). This effect may lead to delayed tillering of main stem leaves, thereby impacting the critical leaf age period for effective tiller initiation and the peak period of stem tillering, increasing the number of missing tiller positions on the main stem and ultimately affecting the panicle number (Huang et al., 2020).

However, it is worth noting that there is a strong compensatory relationship between panicle number and number of spikelet per panicle. When the panicle number is too high, the number of spikelet per panicle may decrease, resulting in no continuous increase in total number of spikelet (Huang et al., 2011). This also explains why, in this study, the total spikelet number and yield were highest under the T2 seeding rate, not under the T1 seeding rate. In summary, the choice of seeding rate significantly influences the yield components and final yield of rice.

### 4.3 Effect of seeding rate on economic benefits

We also analyzed the impact of different seeding rates on economic benefits. Previous research mainly focused on the influence of seeding rates on seedling quality, machine transplanting quality, and yield, but most of them were limited to yield aspects. After conducting a comprehensive investigation on seedling quality, machine transplanting quality, and yield under four seeding rates, this study found that the T1 treatment exhibited the best seedling quality, T3 treatment had optimal machine transplanting quality, and the T2 treatment resulted in the highest yield. However, there was no clear indicator to determine which seeding rate was most suitable for the blanket seedling machine transplanting of Huazheyou 210, thus, we recorded various costs and income in actual production and calculated the final economic benefits for each seeding rate.

Through comparative analysis, we found that as the seeding rate per tray increased, the number of trays required per hectare gradually decreased, reducing the seed usage and consequently lowering the transplanting cost. The results of the two-year experiment showed that the T2 seeding rate performed the best in terms of yield and economic benefits. The T1 seeding rate, due to its higher costs for seedling cultivation and transplanting, resulted in slightly lower economic benefits compared to the lower-yield T3 treatment. As for the T4 seeding rate, although it had the lowest seedling cultivation costs, its yield was too low, resulting in the lowest economic benefits.

Furthermore, by calculating the economic benefits, we derived an equation and determined that the optimal seeding rate was 61.9 grams, which is close to the seeding rate used in the T2 treatment in this experiment. This finding provides crucial reference for the blanket seedling machine transplanting of Huazheyou 210.

## 5 Conclusions

The appropriate seeding rate has a significant impact on the growth and yield of rice. The results of this study indicate that a seeding rate of 60 grams is the most suitable for cultivating blanket seedlings of hybrid rice during machinal transplanting. This seeding rate effectively balances seedling quality and mechanical transplanting quality, ensuring high yield and favorable economic benefits of indica hybrid rice.

## Data availability statement

The raw data supporting the conclusions of this article will be made available by the authors, without undue reservation.

## Author contributions

YF: Writing – original draft, Writing – review & editing. ML: Investigation, Writing – review & editing. KW: Investigation, Writing – review & editing. YL: Investigation, Writing – review & editing. QH: Supervision, Writing – review & editing. HoZ: Supervision, Writing – review & editing. HaZ: Methodology, Supervision, Writing – review & editing.
